# Integrative multi-omics analyses unravel the immunological implication and prognostic significance of CXCL12 in breast cancer

**DOI:** 10.3389/fimmu.2023.1188351

**Published:** 2023-07-26

**Authors:** Zhi-Jie Gao, Zhou Fang, Jing-Ping Yuan, Sheng-Rong Sun, Bei Li

**Affiliations:** ^1^ Department of Breast and Thyroid Surgery, Renmin Hospital of Wuhan University, Wuhan, Hubei, China; ^2^ Department of Pathology, Renmin Hospital of Wuhan University, Wuhan, Hubei, China

**Keywords:** breast cancer, CXCL12, prognostic signature, immune landscape, single-cell RNA-seq, drug screening

## Abstract

**Background:**

CXCL12 is a vital factor in physiological and pathological processes, by inducing migration of multiple cells. We aimed to comprehensively detect the role of CXCL12 in breast cancer, and explore novel CXCL12-related biomarkers through integrative multi-omics analyses to build a powerful prognostic model for breast cancer patients.

**Methods:**

Immunohistochemistry analysis of the tissue microarray was performed to evaluate the correlation between CXCL12 expression levels and breast cancer patient outcomes. Combined single-nucleus and spatial transcriptomics data was used to uncover the expression distribution of CXCL12 in breast cancer microenvironment. CXCL12-related genes were identified by WGCNA analysis. Univariate Cox and LASSO regression analyses were then conducted to screen prognostic genes from above CXCL12-related genes, followed by the construction of the CXCL12-related prognostic signature, identification of risk groups, and external validation of the prognostic signature. Analyses of biological function, mutation landscape, immune checkpoint genes and immune cells, were performed to further reveal the differences between high/low-risk groups. Paired single-cell RNA-seq and bulk RNA-seq were analyzed to further disclose the association between the risk score and the complex tumor immune microenvironment. To screen potential therapeutic agents for breast cancer patients, analyses of gene-drug correlation and sensitivity to immunotherapy were conducted.

**Results:**

High expression of CXCL12 was linked with a prolonged survival in breast cancer. A total of 402 genes were identified by WGCNA analysis and 11 genes, covering *VAT1L, TMEM92, SDC1, RORB, PCSK9, NRN1, NACAD, JPH3, GJA1, BMP8B* and *ADAMTS2*, were screened as the candidate prognostic genes. Next, the prognostic signature was built and validated using these genes to predict the outcomes of breast cancers. The high-risk group patients exhibited significantly inferior prognoses. The combination of the risk score and tumor mutational burden (TMB) had remarkably improved performance in predicting patient outcomes. Besides, high-risk group patients showed higher infiltration of M2-like macrophages. Finally, several potential anticancer drugs were identified. The high-risk group patients were more sensitive to immunotherapy but resistant to docetaxel.

**Conclusions:**

CXCL12 has important immunological implication and prognostic significance in breast cancer. The CXCL12-related prognostic model could well predict the prognosis and treatment response of breast cancers.

## Introduction

Worldwide, breast cancer has become one of the most prevalent malignancies with an estimated 2.3 million new cases in 2020 on the basis of the latest global cancer statistics (1). Accounting for around 30% of female cancers, breast cancer exhibits a mortality-to-incidence ratio of 15% (2). These statistics highlight the urgent need to develop novel effective strategies for breast cancer diagnosis and treatment. The emergence of molecular targeted therapy and immunotherapy has brought new treatment prospects for breast cancer patients. However, not all breast cancer patients respond to the same treatment. For instance, immune checkpoint blockade (ICB) has shown considerable efficacy on numerous malignancies, but is ineffective for most patients owing to primary or acquired resistance (3). In recent years, as precision medicine is becoming a reality, treatments of breast cancer have gradually evolved from empirical chemotherapy to personalized therapies. Thus, an outstanding question is to identify which underpinning mechanisms and related biomarkers determine treatment responses.

Chemokines are a family of small and secreted proteins responsible for regulating directed cell migration (chemotaxis) through binding to Gai-protein-coupled seven-transmembrane-spanning receptors (GPCRs) which are so-called classical chemokine receptors (4). Atypical chemokine receptors (ACKRs) which are also seven-transmembrane-spanning receptors can also interact with chemokines and function as scavenging receptors, but fail to induce chemotaxis (5). Additionally, chemokines exert an important role in cell survival, proliferation and differentiation, tumor growth and metastasis, as well as angiogenesis, hematopoiesis, organogenesis, and multiple other processes. Based on the relative location of the conserved cysteine residues in their amino acid sequence, chemokines could be classified as four subfamilies: C, CC, CXC, and CX3C. Heretofore, nearly 50 different chemokine ligands, as well as 20 diverse signaling GPCRs and 4 ACKRs have been characterized in humans. Biologically, chemokines are divided into inflammatory and homeostatic chemokines based on their expression and function. Induced by response to endogenous and exogenous inflammatory triggers, inflammatory chemokines are rapidly secreted and can coordinate the directional chemotaxis of leukocytes to inflammatory sites. Homeostatic chemokines are constitutively expressed and regulate homeostatic basal migration of immune cells at physiological conditions. In this way, chemokines and their receptors play a key role in the positioning of cell populations both in regular physiological processes and abnormal conditions like infection and particularly, in cancer.

Initially discovered as pre-B cell growth factor (PBGF), the CXC chemokine CXCL12 was found to be indispensable for homeostatic processes such as embryogenesis and lymphopoiesis. Subsequently, PBGF was found to express constitutively in bone marrow stromal cells and was therefore named stromal cell-derived factor-1 (SDF-1). CXCL12 is mostly considered as a homeostatic chemokine which induces the migration and activation of hematopoietic progenitor cells, endothelial cells, and multiple leukocytes. However, CXCL12 can also play an inflammatory function in particular conditions by the synergy with CXCL8 and other chemokines to induce chemotaxis of leukocytes (6). Under homeostatic and pathological conditions, CXCL12 implements its functions by the interaction with its receptors CXC chemokine receptor CXCR4 and atypical chemokine receptor ACKR1 and ACKR3 or by binding to glycosaminoglycans (GAGs) in tissues and endothelium to present to leukocytes (7). The CXCR4-CXCL12 chemokine axis is found to be important in the preservation of immature and mature immune cells within the bone marrow (4). In addition, the CXCR4-CXCL12 axis promotes the differentiation of newly recruited tumor-associated macrophages (TAMs) into perivascular TAMs to accelerate vascular leakiness and tumor cell intravasation (8). Apart from the function in chemotaxis, CXCL12 can also facilitate CD4^+^ T cell survival by binding to CXCR4 and subsequently activating the PI3K and MAPK signaling pathways (9). In breast cancer preclinical models, high expression of CXCL12 is linked with a prolonged disease-free survival (DFS) and overall survival (OS), presumably owing to decreased metastasis of breast cancer cells (10). Furthermore, a systematical meta-analysis study has revealed that breast cancer patients with high CXCL12 expression harbor an OS advantage (11). Nevertheless, it is still not straightforward to delineate the complex role of CXCL12 and CXCL12-related biomarkers in the diagnosis and treatment of breast cancers.

In this study, we systematically investigated the association between the expression of CXCL12 and clinical characteristics in breast cancer, and found that CXCL12 was lower expressed in the tumor tissues and linked with a survival advantage. Besides, we performed immunohistochemistry (IHC) analysis to validate that breast cancer patients with higher expression of CXCL12 possess preferred outcomes. To further explore the architectural feature of CXCL12-expressing cell populations and their co-location with other cell populations, we conducted spatially resolved transcriptomics analysis with paired single-nucleus RNA-seq (snRNA-seq), which could determine how diverse cells in the tumor microenvironment (TME) are organized as functional units. The most significantly related gene module of CXCL12 was identified via Weighted Gene Co-expression Network Analysis (WGCNA). Next, we constructed a prognostic signature by carrying out univariate Cox regression and least absolute shrinkage and selection operator (LASSO) regression analyses in The Cancer Genome Atlas (TCGA) breast cancer training cohort. After multiple additional validation cohorts, we demonstrated that this CXCL12-related signature could predict the prognoses of breast cancers. In addition, the differences in pathway activities, mutational and immune statuses were explored between the high- and low-risk groups. Considering single-cell RNA-seq (scRNA-seq) allowed us to study the heterogeneity of tumors at the single-cell resolution, we made full use of a public scRNA-seq dataset with paired bulk RNA-seq data and revealed distinct composition of cell subpopulations in different risk group samples. We proved that the M2-like macrophages played a vital role in the high-risk group patients via deconvolution and scRNA-seq analyses. Responses to immunotherapy and sensitivities to multiple anti-cancer drugs were also predicted based on our risk groups. Finally, we built a nomogram to provide guidance for breast cancer treatment. Summarily, this study revealed the immunological implication and prognostic significance of CXCL12 in breast cancer, and that its related biomarkers might provide us with a novel research direction for the diagnosis and treatment of breast cancer.

## Materials and methods

### Collection of public datasets

RNA-sequencing expression matrix and clinical information of normal breast samples and breast cancer samples from the Cancer Genome Atlas (TCGA) database were downloaded from cBioportal (https://www.cbioportal.org/). Two additional datasets (GSE42568 and GSE183947) containing expression matrix of tumorous and normal breast samples were obtained from the GEO database (https://www.ncbi.nlm.nih.gov/geo/). We downloaded somatic mutation data from Genomic Data Commons (GDC, https://portal.gdc.cancer.gov/). Somatic mutation data sorted in the form of Mutation Annotation Format (maf) were analyzed and then used to calculate TMB using the R package maftools.

To validate the predictive power of the CXCL12-related prognostic model, we downloaded three additional independent datasets from the GEO database. After eliminating pathologies with duplicate and incomplete survival information, three validation sets were obtained, including GSE19615 with 115 samples, GSE21653 with 252 samples, and GSE61304 with 58 samples.

### Immunohistochemistry

We collected the tissue microarray (TMA) containing a total of 157 human breast cancer specimens from the People’s Hospital of Wuhan University for pathological analysis of CXCL12. IHC staining was performed as follows: deparaffinization, antigen retrieval, blocking endogenous peroxidase (3%hydrogen peroxide solution, room temperature, out of light for 25 minutes), serum block (3% bovine serum albumin, room temperature, 30 min), primary antibodies of CXCL12 (Abcam, ab9797,1/200) were incubated overnight at 4 °C, and horseradish peroxidase (HRP)-conjugated for 50 minutes at room temperature. Staining was visualized with DAB and time controlled under a microscope. Finally, nuclear counterstaining was performed using hematoxylin for approximately 3 minutes. Human breast cancer specimens treated with only secondary antibody served as negative controls and paraffin-embedded human hepatic carcinoma specimens were used as positive controls. The staining results were scored by two independent pathologists as follows: the protein expression levels of CXCL12 were described by the percentage of positive cells calculated by ImageJ software.

### Acquisition of CXCL12-related genes

We analyzed breast cancer expression data using the Weighted Gene Co-expression Network Analysis (WGCNA) package to obtain genes most related to CXCL12. Samples were clustered to ascertain the overall relevance of all samples in the dataset, and outliers were excluded. The soft thresholding power β was chosen based on the lowest power for which the scale-free topology fit index reached a high value. The minimum gene number/module was set to 50 and, subsequently, 8 modules were generated. Besides, we conducted correlation analyses between modules and traits to find the most relevant modules for CXCL12, which contained a total of 402 genes.

### Construction and validation of a CXCL12-related prognostic signature

First, RNA expression matrix of the breast cancer samples in TCGA, GSE19615, GSE21653 and GSE61304 datasets was cross-checked to identify co-expressed genes. To obtain CXCL12-related genes that could construct a prognostic signature, univariate Cox regression and least absolute shrinkage and selection operator (LASSO) regression analyses were carried out. We eventually obtained 11 genes, including *VAT1L, TMEM92, SDC1, RORB, PCSK9, NRN1, NACAD, JPH3, GJA1, BMP8B* and *ADAMTS2*, and constructed a CXCL12-related prognostic model based on these genes. To group the breast cancer patients, the risk score of each breast cancer patient in the training set was calculated according to the following formula:


Risk score=∑ni=∑(Coefi*xi)

The breast cancer patients were then categorized into the high-risk and low-risk groups according to the median of risk scores. The predictive sensitivity of the risk score was painted via the R package survivalROC for estimation. The model effectiveness was evaluated in the validation set using the same coefficient and cutoff values that were used in the training set.

### Biological functional analysis between high- and low-risk group patients

The Deseq2 R package was used to perform differentially expressed genes (DEGs) analysis. DEGs were determined with a cutoff of an adjust p-value of less than 0.05 and |Log2 fold change| greater than 1. The clusterProfiler R package was used to perform gene set enrichment analysis (GSEA). With the use of Fisher’s exact test, those with false discovery rate FDR-corrected p-values of less than 0.05 were regarded as marked indicators. Gene set variation analysis (GSVA) was performed via the R package GSVA. Gene signatures of recurrent cancer cell states were collected from the previous study (12).

### Tumor immune microenvironment in breast cancer patients

In order to study the infiltration of immune cells, we used TIMER2.0, an efficient algorithm for predicting immune cell infiltration of bulk tumor gene expression data (http://timer.cistrome.org/). For each sample, CIBERSORT quantified the relative abundance of 22 immune cells. In addition, we collected series of tumor immunomodulators from the literatures and calculated the correlation of risk score with them.

### Single-cell transcriptome analysis

First, we downloaded scRNA-seq data and paired bulk RNA-seq data 24 breast tumors from the GEO (GSE176078) database. We conducted unsupervised clustering of the single cells using the read count matrix as input via Seurat package (v4.1.1) in R (v4.1.3). The quality control was applied strictly and was mainly based on the number of detected genes and proportion of mitochondrial gene count per cell. At first, cells with less than 200 detected genes and cells with over than 15% mitochondrial gene count were filtered. Aiming to avoid unexpected noise, genes detected in less than 3 cells were excluded from the downstream analysis. We performed the fast mutual nearest neighbor (fastMNN) algorithm to integrate multi-sample data and correct the batch effect via Seurat-Wrappers package (v0.3.0). Subsequently, we performed dimension reduction clustering and differential expression analysis following the Seurat-guided tutorial. The principal component analysis (PCA) and uniform manifold approximation and projection (UMAP) dimension reduction were performed with the top 15 principal components. The annotation of cell clusters was based on the canonical gene markers. The signature gene list of M2-like macrophages has been previous described (13).

### Combined single-nucleus and spatial transcriptome sequencing and analysis

The detailed pipeline of nuclei isolation, single-nucleus RNA-sequencing (snRNA-seq) and spatial transcriptomics was according to our previous study (14). Strict data quality control and downstream dimension reduction clustering were performed similarly to the above described scRNA-seq data processing. We conducted data normalization on the independent tissue sections via the variance-stabilizing transformation method implemented in the SCTransform function in Seurat package. Furthermore, we mapped the distribution of cancer-associated fibroblasts (CAFs) in the breast cancer tissue using the top 50 gene markers based on the snRNA-seq data, by the single-sample gene set enrichment analysis (ssGSEA).

### Predicting drug responses and immunotherapy sensitivity

We used the R package oncoPredict to assess the predictive ability of risk score chemotherapeutic agents by calculating patients IC50 for various common chemotherapeutic agents. The Wilcoxon rank test was then used to compare the difference in IC50 between the high/low-risk groups. To further validate the predictive value of above immunotherapy responses, we used the extra immunotherapy data set IMvigor210 (uroepithelial carcinoma) to predict immunotherapy response.

### Univariate and multivariable Cox regression

We performed univariate Cox regression on breast cancer patients with gene expression and overall survival. Multivariate Cox regression was used to evaluate independent risk factors in the same cohort. Genes and factors with a false discovery rate (FDR) < 0.05 were considered statistically associated with patient survival. The results of univariate and multivariate Cox regression were acquired and visualized by using the R package forestplot.

### Establishment of the nomogram

This study used the Cox regression model along with the R package rms to build an OS prediction nomogram that set 1-, 3-, and 5-year OS as the endpoints. The C-index was used to estimate the discriminative ability of the nomogram. Calibration plots were used to visualize the consistency between the predicted and factual 1-, 3-, and 5-year OS.

### Statistical analysis

All statistical analyses were performed using R version 4.1.3 (https://www.r-project.org/) and its adequate packages. Statistical significance was set at p ≤ 0.05.

## Results

### Analysis of the correlation between clinical characteristics and CXCL12 in breast cancer

**Figure 1:**
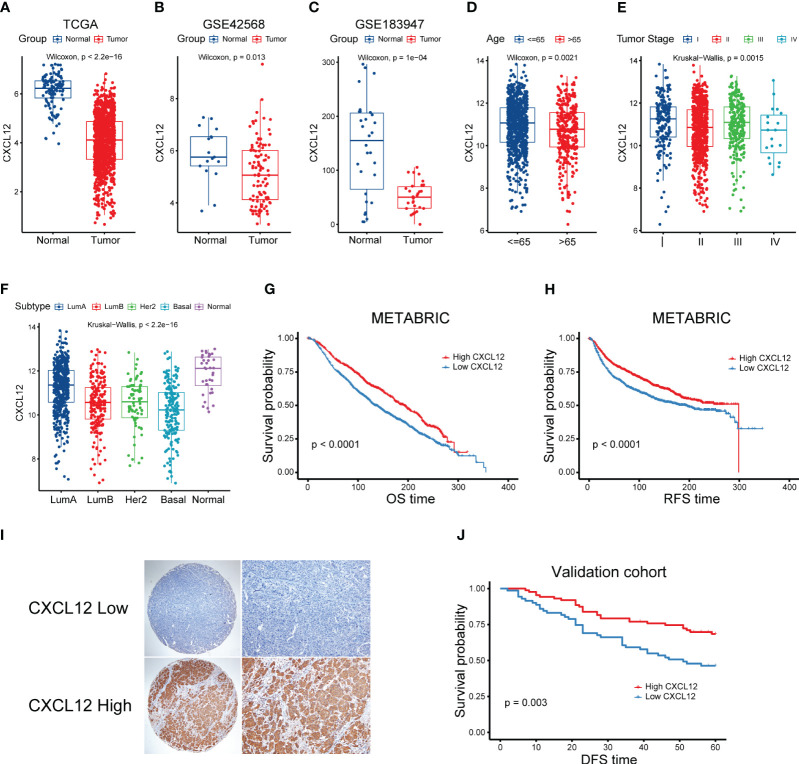
Correlation between clinical characteristics and CXCL12 in breast cancer. **(A)** Expression levels of CXCL12 mRNA of breast cancers in the TCGA cohort. **(B, C)** Expression levels of CXCL12 mRNA of breast cancers of two GEO datasets. **(D–F)** Association between CXCL12 and age, tumor stage and molecular subtype of breast cancers in the TCGA breast cancer cohort. **(G)** Kaplan-Meier survival analysis was performed on the relationship between CXCL12 and OS using the METABRIC cohort. **(H)** Kaplan-Meier survival analysis was performed on the relationship between CXCL12 and RFS using the METABRIC cohort. **(I)** Representative immunohistochemical staining of CXCL12 protein in breast cancer tissue microarrays. **(J)** Kaplan-Meier survival analysis was performed on the relationship between CXCL12 and DFS using our validation cohort.

To explore the complex role of CXCL12 in breast cancer, we first evaluated the expression levels of CXCL12 in breast cancer tissues and non-tumor tissues in the TCGA breast cancer cohort. It was revealed that the mRNA levels of CXCL12 were markedly reduced in tumorous tissues (**Figure 1A**). Additionally, we further validated the expression levels of CXCL12 within normal and tumorous breast samples from two GEO datasets and found similar results (**Figures 1B, C**). Next, we showed that CXCL12 expression level was lower in the aged population, implying the vital implication during aging (**Figure 1D**). Besides, we found that CXCL12 was highly expressed in breast cancer tissues from patients with stage I, compared to those with stage II, III and IV disease (**Figure 1E**). Among diverse PAM50 molecular subtypes, CXCL12 was strikingly higher in the normal-like subtype, which is only a small subset of breast cancers (**Figure 1F**). CXCL12 expression levels in the basal-like subtype were significantly lower than those in luminal A/B and human epidermal growth receptor 2 (HER-2)-enriched tumors (**Figure 1F**). In addition, it is indicated that CXCL12 expression was substantially associated with aneuploidy and microsatellite instability (MSI) in breast cancers (**Supplementary Figures 1A, B**). We further depicted the landscape of somatic mutations in two groups according to the median expression level of CXCL12, and found more *PIK3CA* and *CDH1*, but less *TP53* mutations in the CXCL12-high group patients (**Supplementary Figure 1C**). In order to figure out the correlation between the CXCL12 expression and immune cell infiltration, we performed CIBERSORT algorithm and found that CXCL12 was positively related to the infiltration of memory resting CD4^+^ T cells, gamma delta T cells, CD8^+^ T cells, and activated mast cells (**Supplementary Figure 1D**).

### Prognostic potential of CXCL12 in breast cancer

Next, we aimed to elucidate the prognostic potential of CXCL12 expression in breast cancer, we first explored the Molecular Taxonomy of Breast Cancer International Consortium (METABRIC) cohort, which was the largest breast cancer cohort so far. We found that high expression of CXCL12 was significantly linked with superior OS and recurrence-free survival (RFS) of breast cancers (**Figures 1G, H**). Considering different expression levels of CXCL12 among diverse molecular subtypes of breast cancer, we conducted the survival analyses among every subtype. We found that high expression level of CXCL12 correlated with a better survival among luminal A/B and normal-like subtype breast cancer patients (**Supplementary Figures 2A–C**). However, patients with high or low expression levels of CXCL12 among the basal-like and HER2-enriched subtypes failed to exhibit a different outcome (**Supplementary Figures 2D, E**). To further validate these results, we performed IHC staining on the tissue microarray (TMA) and survival analysis in independent breast cancer samples. As expected, CXCL12 expression was cytoplasmic (**Figure 1I**). Our validation analysis showed that higher CXCL12 expression level correlated to superior disease-free survival (DFS) in breast cancer (**Figure 1J**).

### Combined snRNA-seq and spatial transcriptome analysis reveal the expression distribution of CXCL12 in breast cancer

**Figure 2:**
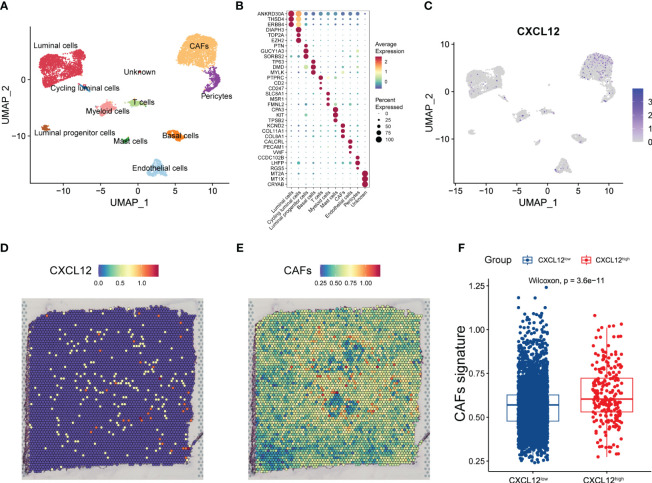
Combined analysis of snRNA-seq and spatial transcriptomics reveals the expression pattern of CXCL12 in breast cancer. **(A)** UMAP plot showing the major cell subpopulations in breast cancer. **(B)** Bubble heatmap showing expression levels of selected signature genes in breast cancer. Dot size indicates fraction of expressing cells, colored based on normalized expression levels. **(C)** UMAP plot showing the expression of CXCL12 in breast cancer. **(D)** The spatial image reveals the expression distribution of CXCL12 in breast cancer. **(E)** Scaled deconvolution values for CAFs overlaid onto tissue spots. **(F)** Boxplot showing the signature score of CAFs in CXCL12^high^ and CXCL12^low^ spots. Paired two-sided Wilcoxon test.

Given the prognostic implication of CXCL12 for breast cancer, we further devoted to uncovering the expression pattern of CXCL12 in the complex cellular clusters and architecture. As reported before, we performed snRNA-seq and sample-paired microarray-based spatial transcriptome combined analyses (14). For the snRNA-seq, we identified three epithelial lineages including mature luminal cells, luminal progenitors and basal cell, and diverse immune and stromal cell clusters including T cells, myeloid cells, mast cells, cancer-associated fibroblasts (CAFs), endothelial cells and pericytes (**Figure 2A**). Based a series of classical gene markers, we annotated these cell clusters minutely (**Figure 2B**). Next, we profiled the expression of CXCL12 by snRNA-seq via the uniform manifold approximation and projection (UMAP) algorithm, and showed that CXCL12 was mainly expressed in the CAFs (**Figure 2C**). Spatially, we mapped CXCL12 and CAFs in the breast cancer tissue, and found overlapped regions in the spatial capture locations (**Figures 2D, E**). Furthermore, we revealed that the CXCL12^high^ regions exhibited higher CAFs signature score which was calculated by single-sample gene set enrichment analysis (ssGSEA) (**Figure 2F**). Summarily, the combined sample-paired snRNA-seq and spatial transcriptome analysis uncovers the expression pattern of CXCL12 in the breast cancer tissue.

### Construction of a CXCL12-related prognostic signature in breast cancer

**Figure 3:**
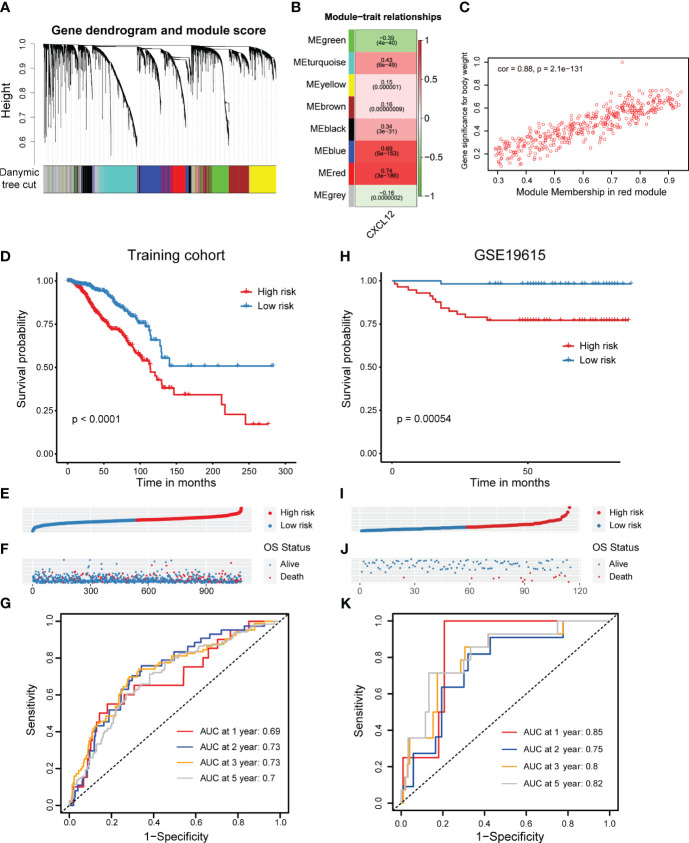
Screening of CXCL12-related genes and construction as well as validation of a CXCL12-related prognostic signature in breast cancer. **(A, B)** Correlation analysis of modules with traits yielded eight non-gray modules, with the red module considered to be the most relevant module for CXCL12. **(C)** Scatter plot of the red module. **(D)** Kaplan-Meier survival analysis was performed on the relationship between the risk score and OS using the TCGA training cohort. **(E)** The rank of risk scores in the TCGA training cohort. **(F)** Survival status in the TCGA training cohort. **(G)** Time-dependent ROC curve analysis of the prognostic model (1, 2, 3, and 5 years) in the TCGA training cohort. **(H)** Kaplan-Meier survival analysis was performed on the relationship between the risk score and OS using the GSE19615 validation cohort. **(I)** The rank of risk scores in the GSE19615 validation cohort. **(J)** Survival status in the GSE19615 validation cohort. **(K)** Time-dependent ROC curve analysis of the prognostic model (1-, 2-, 3-, and 5-year) in the GSE19615 validation cohort.

Next, we looked forward to further clarify the prognostic potential of CXCL12-realted genes in breast cancer. Firstly, we conducted WGCNA analysis and obtained eight gene modules through average hierarchical clustering and dynamic tree clipping (**Figure 3A**). To pick out the key modules, the relationship between the modules and CXCL12 was studied. Among these eight gene modules, the red module was strikingly correlated with the expression of CXCL12 (**Figure 3B**). Thus, the red gene module was selected as the most important module related to CXCL12 for further analysis. It was shown that significant correlation existed between the module membership and gene significance of the red module (**Figure 3C**). Finally, 402 genes in the red module were enrolled for downstream analyses (**Supplementary Table 1**).

Initially, the univariate Cox regression analysis revealed that 19 genes were linked with the breast cancer prognosis (**Supplementary Table 1**). Subsequently, we carried out the LASSO analysis and identified 11 prognostic signature genes, including *VAT1L, TMEM92, SDC1, RORB, PCSK9, NRN1, NACAD, JPH3, GJA1, BMP8B* and *ADAMTS2* (**Supplementary Figures 3A, B**). According to the coefficients and expression levels of these prognostic signature genes, we calculated the risk score of each sample in the TCGA breast cancer cohort, which was our training set. Then, we divided the breast cancer patients among the TCGA training cohort into high- and low-risk groups based on the median risk score, and found that the low-risk group patients had a significantly superior outcome (**Figures 3D–F**). To evaluate the performance of this risk model, we showed the receiver operator characteristic (ROC) curves and found the area under the ROC curve (AUC) value at 1, 2, 3, 5 years was 0.69, 0.73, 0.73, 0.7, respectively (**Figure 3G**). Moreover, we detected the association between the risk score and clinical features such as the clinical tumor stage (I-IV) and molecular subtype. It was showed that the risk score was significantly higher in the stage III and IV patients, implying this CXCL12-related risk score correlated to the progression of breast cancer (**Supplementary Figure 3A**). Next, considering the very limited number of patients in stage IV, we only investigated the prognostic role of our risk score among stage I-III. We found patients with a higher risk score had an evidently improved survival in stage II and III, but not significantly in stage I (**Supplementary Figures 3B–D**). Additionally, we exhibited this risk score was highest in the HER2-enriched subtype patients, but lowest in the normal-like subtype patients (**Supplementary Figure 3E**). Similarly, we decided not to conduct the survival analysis among normal-like subtype patients, because of the small number of cases. Elsewhere, we exhibited that patients with a higher risk score harbored an inferior prognosis in luminal A/B and basal-like subtypes, expect for HER2-enriched subtype (**Supplementary Figures 3F-I**).

In order to verify the reliability of the CXCL12-related prognostic signature, we further performed validations in several additional validation sets. Similarly, we grouped breast cancer patients in the GSE19615 cohort by the median risk score, which was coordinate with the training set, and also figured out that high-risk group patients obtained an inferior prognosis (**Figures 3H-J**). The AUC values of risk score in GSE19615 dataset were 0.85 for 1-year, 0.75 for 2-year, 0.8 for 3-year and 0.82 for 5-year (**Figure 3K**). Besides, breast cancer patients with a higher risk score in the GSE21653 and GSE61304 datasets also showed significantly shorter survival (**Supplementary Figures 2C-J**). Taken together, we built and validated a novel CXCL12-related prognostic signature for predicting the outcomes of breast cancers.

### Functional and genomic features of CXCL12-related risk score-based classification

**Figure 4:**
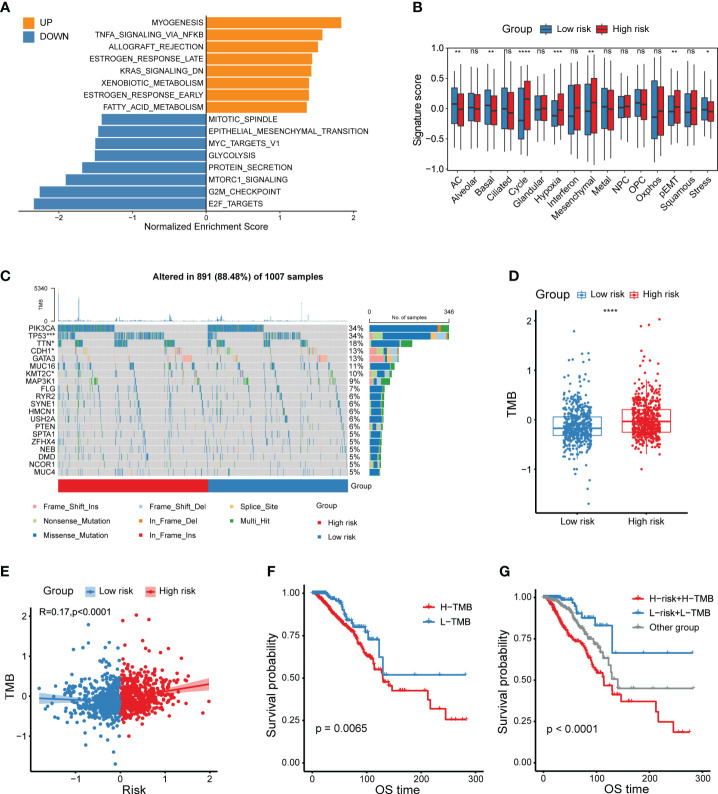
Functional and genomic features of CXCL12-related risk score-based classification. **(A)** Bar plot showing different pathways enriched in high/low-risk groups of breast cancer calculated by GSEA. **(B)** Boxplots showing the signature score of 16 cancer cell states in high/low-risk groups of breast cancer scored by GSVA. Paired two-sided Wilcoxon test. **(C)** Waterfall plot represents the mutation distribution of the most frequently mutated genes in high/low-risk groups. **(D)** Boxplot showing the levels of TMB in high/low-risk groups. Paired two-sided Wilcoxon test. **(E)** Scatter plot showing the correlation between the risk score and TMB in the TCGA breast cancer cohort. **(F)** Kaplan-Meier survival analysis was performed on the relationship between TMB and OS in the TCGA breast cancer cohort. **(G)** Kaplan-Meier survival analysis was performed on the relationship between combination of TMB and the risk score and OS in the TCGA breast cancer cohort. The asterisks represent the statistical P value (*p<0.05; **p<0.01; ***p <.001; ****p < 0.0001; ns p>0.05).

With the intention to delve the underpinning mechanisms explaining the prognostic implication of this CXCL12-related risk score, we first committed to explore the functional and genomic features. Gene set enrichment analysis (GSEA) analysis revealed that breast cancer patients in the high-risk group showed a considerably enrichment in myogenesis, TNFα signaling via NF-κB as well as early and late estrogen response (**Figure 4A**, **Supplementary Figures 5A-C**). However, E2F targets, G2M checkpoint, MTORC1 signaling and MYC targets pathways were enriched in the low-risk group patients (**Figure 4A**, **Supplementary Figures 5D, E**). To obtain insights into the transcriptional heterogeneity among breast cancer patients, we performed gene set variation analysis (GSVA) algorithm to calculate 16 recurrent cancer cell states which interacted with the TME to take shape organized systems qualified to promote immune escape, metastasis and drug resistance (12). We uncovered patients in the high-risk group showed a higher signature score of cycling, hypoxia, mesenchymal, partial epithelial-mesenchymal transition (pEMT) and stress (**Figure 4B**). However, astrocyte (AC)-like and basal modules were found to be enriched among low-risk group patients (**Figure 4B**).

Besides, we depicted and compared the mutation features of high- and low-risk group patients, and showed higher mutation frequency of *TP53*, *TTN* and *KMT2C* in high-risk group, but lower mutation frequency of *CDH1* in the low-risk group (**Figure 4C**). Considering that tumor mutation burden (TMB) was already well known to predict the response to immunotherapy, we aimed to compare the TMB levels between different risk groups. Obviously, high-risk group patients exhibited a higher TMB level (**Figure 4D**). In addition, the risk score was positively correlated with the TMB level (**Figure 4E**). As expected, patients with higher TMB levels had a relatively shorter survival, which was not enough significant (**Figure 4F**). Therefore, we investigated whether the combination of TMB and the CXCL12-related risk score could jointly stratify breast cancer patients into groups with conspicuously distinct prognoses. Consequently, our work supported that the joint stratification of TMB and CXCL12-related risk score could be used to categorize breast tumors into distinct outcomes (**Figure 4G**).

### Dissection of tumor immune microenvironment features based on CXCL12-related prognostic signature

**Figure 5:**
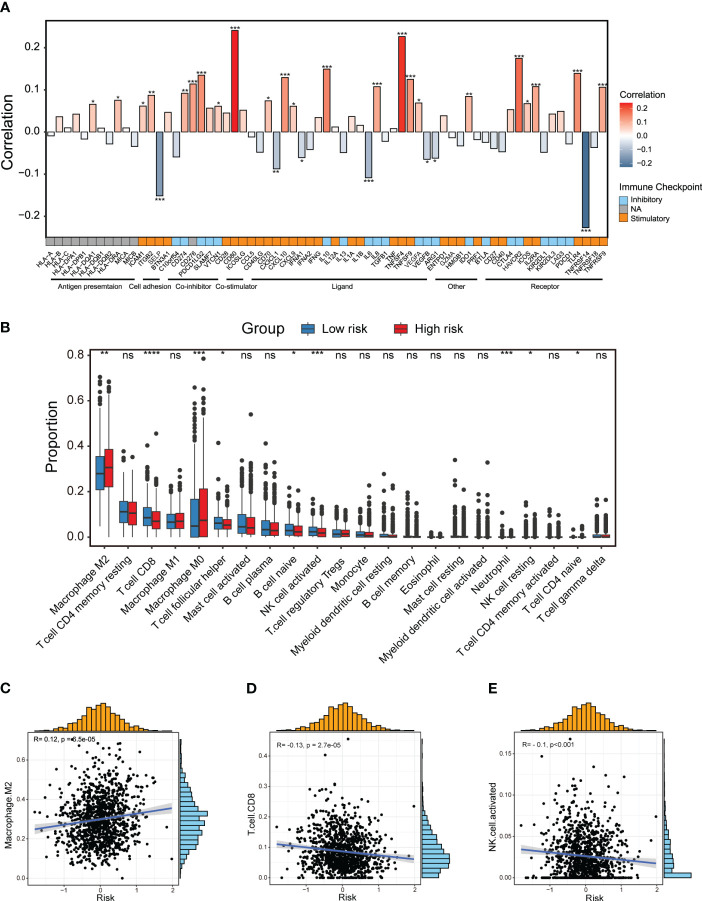
Dissection of tumor immune microenvironment features based on the CXCL12-related prognostic signature. **(A)** Bar plot of the correlation between immunomodulators and the risk score in the TCGA breast cancer cohort. **(B)** Boxplots showing the proportion of 22 immune cells in high/low-risk groups of breast cancer estimated by CIBERSORT. Paired two-sided Wilcoxon test. **(C–E)** Scatter plots showing the correlation between the risk score and the proportion of M2-like macrophages, CD8+ T cells and activated NK cells in the TCGA breast cancer cohort. The asterisks represent the statistical P value (*p<0.05; **p<0.01; ***p <.001; ****p < 0.0001; ns p>0.05).

Next, we aimed to uncover differences in the tumor immune microenvironment (TIME) between two risk groups. Firstly, we explored that there was a distinct association between immunomodulators and the CXCL12-related risk score as illustrated by the bar plot (**Figure 5A**). In particular, the risk score showed significantly positive correlation with inhibitory immune checkpoint markers *HAVCR2* and *PDCD1LG2*, while negative correlation with stimulatory immune checkpoint markers *SELP* and *TNFRSF14* (**Figure 5A**). Then, we performed CIBERSORT algorithms to evaluate the enrichment level of diverse immune cells (**Figure 5B**). Dramatically, the M2-like macrophage was enriched in the high-risk group patients, and its enrichment score was positively linked with the CXCL12-related risk score (**Figures 5B, C**). On the other hand, we observed significantly abundant infiltration of CD8^+^ T cells in the low-risk group patients (**Figures 5B, D**). The activated natural killer (NK) cells were also found to be enriched in the low-risk group, and negative correlated with the risk score (**Figures 5B, E**).

**Figure 6:**
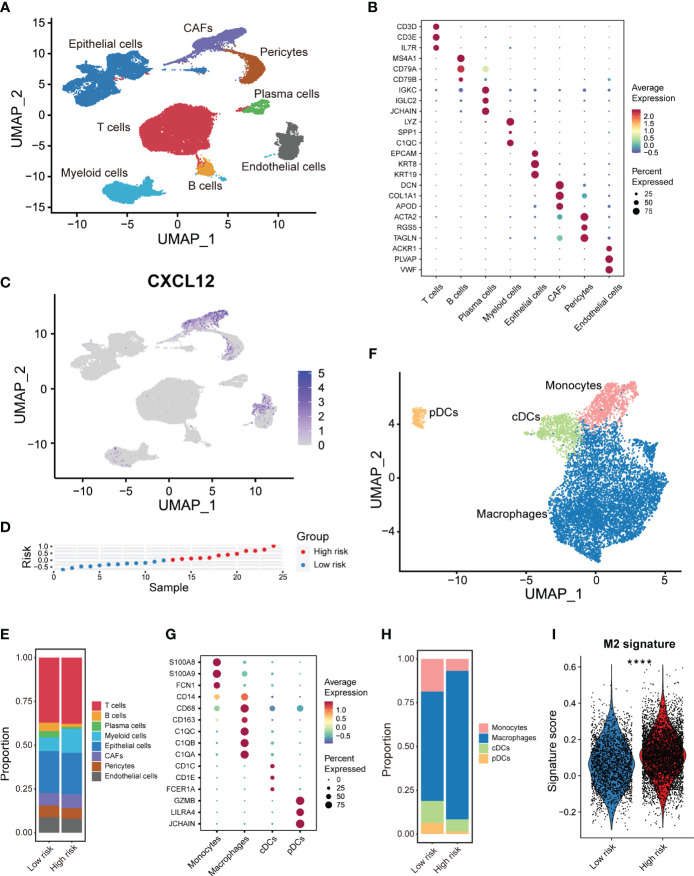
scRNA-seq analysis of the tumor immune microenvironment features based on the CXCL12-related prognostic signature. **(A)** UMAP plot showing the major cell subpopulations in breast cancers. **(B)** Bubble heatmap showing expression levels of selected signature genes in breast cancers. Dot size indicates fraction of expressing cells, colored based on normalized expression levels. **(C)** UMAP plot showing the expression of CXCL12 in breast cancer. **(D)** The rank of risk scores based on the bulk RNA-seq expression in the GSE176078 cohort. **(E)** Relative proportions of diverse cell types across high/low-risk tumors. **(F)** UMAP plot showing the diverse subsets of myeloid cells in breast cancers. **(G)** Bubble heatmap showing expression levels of selected signature genes for myeloid cells in breast cancers. Dot size indicates fraction of expressing cells, colored based on normalized expression levels. **(H)** Relative proportions of diverse subpopulations of myeloid cells across high/low-risk tumors. **(I)** Boxplot showing the M2-like macrophage signature scores in the macrophage subset of high/low-risk tumors. Paired two-sided Wilcoxon test. The asterisks represent the statistical P value (****p < 0.0001).

Furthermore, determining to minutely explore distinct cellular composition in the high- and low-risk patients at single-cell resolution, we made full use of the public scRNA-seq with paired bulk RNA-seq data of breast cancers (15). We integrated 24 samples with paired bulk and scRNA-seq data and corrected the batch effects via mutual nearest neighbor (MNN) algorithm. By strict quality control within every single sample, we depicted the breast cancer cellular landscape at single-cell resolution by UMAP (**Figure 6A**). Using canonical lineage markers, we annotated each cell population as epithelial cells, T cells, B cells, plasma cells, myeloid cells, endothelial cells, pericytes and CAFs (**Figure 6B**). For instance, T cells were identified based on the expression of *CD3D*, *CD3E* and *IL7R* (**Figure 6B**). Besides, we annotated the epithelial cells due to the unique expression of *EPCAM*, *KRT8* and *KRT19* (**Figure 6B**). Obviously, *CXCL12* was mainly expressed in the CAFs and a subset of pericytes and endothelial cells (**Figure 6C**). We also detected the expression of *CXCR4*, which was one of the main receptors of CXCL12, was considerably higher in the immune cells including T, B and myeloid cells (**Supplementary Figure 6A**). This result implied the important role of CXCR4-CXCL12 axis in the immune-stromal interaction of breast cancer. Besides, *ACKR3* was scarcely expressed, and *ACKR1* was expressed in a subset of endothelial cells in breast cancer (**Supplementary Figures 6B, C**).

Additionally, we classified these 24 samples into high- and low-risk group based on the median value of the calculated risk score (**Figure 6D**). High/low-risk group samples exhibited a distinct UMAP plot, especial for the epithelial cells (**Supplementary Figures 6D, E**). We observed distinct cellular composition in two groups (**Figure 6E**). Particularly, the enrichment of the myeloid cells which generally exerted an immunosuppressive role, was much higher in the high-risk samples (**Figure 6E**). This finding was coordinated with our above immune cell infiltration analysis by CIBERSORT. To acquire deeper insights into the myeloid cells, we further re-clustered the myeloid cells, and identified monocytes, macrophages, conventional dendritic cells (cDCs), and plasmacytoid DCs (pDCs) based on multiple cell markers (**Figures 6F, G**). It was shown that the composition of macrophages was significantly higher in the high-risk group patients (**Figure 6H**). Afterwards, we calculated the signature score of the immunosuppressive M2-like macrophage in the macrophage subset of myeloid cells. We revealed that the immunosuppressive M2-like macrophage signature score was markedly higher in the high-risk group patients, which was anastomotic with the CIBERSORT results (**Figure 6I**). Above results showed obvious differences of immune features between two risk groups, and that the infiltration of M2-like macrophages might chiefly contribute to the poor prognosis of high-risk breast cancers.

### High- and low-risk group patients differ in drug sensitivity and response to immunotherapy

**Figure 7:**
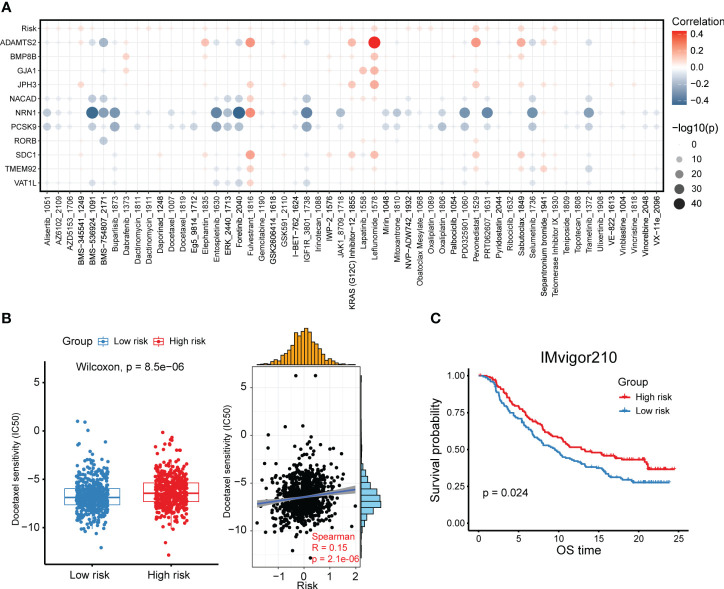
High- and low-risk group patients differ in drug sensitivity and response to immunotherapy. **(A)** Bubble plot showing the relationship between drugs, risk score, and model genes. **(B)** Boxplot showing the comparison of IC50 of docetaxel between high- and low-risk groups, and scatter plot showing the correlation between the IC50 of drugs and the risk score in the TCGA breast cancer cohort. **(C)** Kaplan-Meier survival analysis was performed on the relationship between the risk score and OS in the IMvigor210 immunotherapy cohort.

A significant implication of tumor heterogeneity is the truth that distinct group of patients respond differently to treatments, promoting treatment failure and recurrence. Aiming to figure out the relationship between the risk score and drug sensitivity, we calculated the half maximal inhibitory concentration (IC50) value of each drug in breast cancer patients in TCGA. The landscape of the significance and association between the drug sensitivities and the risk score as well as the candidate prognostic genes was detected among two groups (**Figure 7A**). We found the IC50 values of leflunomide, pevonedistat, sabutoclax and telomerase inhibitor IV were positively correlated with the risk score (**Figure 7A**). Besides, *ADAMTS2* was conspicuously positively related to the IC50 of leflunomide, indicating that leflunomide was more suitable for low-risk group patients (**Figure 7A**). On the other hand, several drugs such as BMS-536924, foretinib and PRT062607 were more sensitive to patients with a higher risk score (**Figure 7A**). Furthermore, we compared the IC50 levels of a series of FDA-approved drugs for breast cancer between high- and low-risk group patients. It was suggested that the IC50 levels of docetaxel, mitoxantrone, palbociclib, vincristine, vinorelbine and fulvestrant were higher in the high-risk group (**Figure 7B**, **Supplementary Figures 7A–E**). These results revealed that breast cancer patients with a higher risk score might be sensitive to such FDA-approved drugs. Considering the association between the CXCL12-related risk score and immunosuppressive checkpoint molecules, we committed to explore the association between the risk score and response to immunotherapy, which worked mainly by blocking immunosuppressive checkpoint molecules and reactivating effector T cells to enhance anti-tumor immunity. The patients with a higher risk score exhibited a superior survival in the immunotherapy cohort (**Figure 7C**). Taken together, the above findings revealed that the CXCL12-related risk score could be a reliable tool for predicting drug sensitivity and immunotherapy response in breast cancer patients.

### Construction of a nomogram to forecast survival for breast cancer

**Figure 8:**
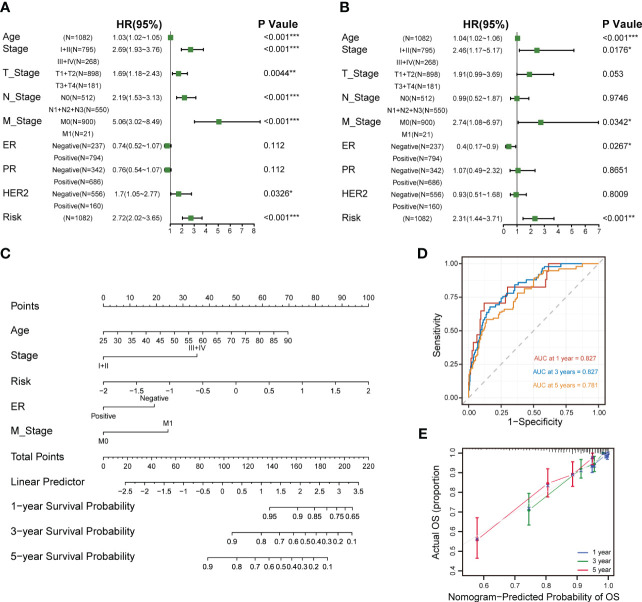
Establishment and assessment of the nomogram survival model. **(A)** Univariate analysis for the clinicopathologic characteristics and the risk score in TCGA cohort. **(B)** Multivariate analysis for the clinicopathologic characteristics and the risk score in the TCGA breast cancer cohort. **(C)** A nomogram was established to predict the prognostic of breast cancers. **(D)** Time-dependent ROC curve analysis of the nomogram (1-, 3-, and 5-year) in the TCGA breast cancer cohort. **(E)** Calibration plots showing the probability of 1-, 3-, and 5-year OS in the TCGA breast cancer cohort (*p<0.05; **p<0.01; ***p <.001; ****p < 0.0001; ns p>0.05).

Univariate and multivariate Cox analyses revealed that age, tumor stage, metastasis stage and the CXCL12-related risk score were independent prognostic factors for breast cancer patients (**Figures 8A, B**). Next, we constructed a predictive nomogram to ameliorate the prognosis efficacy of the CXCL12-related risk score model and to provide a quantitative and visualization tool for predicting 1-, 3-, and 5-year OS (**Figure 8C**). Subsequently, the AUCs of the 1-, 3-, and 5-year OS for the nomogram were 0.827, 0.827, and 0.781, respectively (**Figure 8D**), which showed prominently superior to the prognostic capacity of the CXCL12-related risk score alone. Besides, we depicted the calibration curves to assess the performance of the nomogram, which indicated that the prediction curves of the model were close to the ideal curve (**Figure 8E**). These results revealed that the nomogram model exhibited a critical prediction efficacy for breast cancer patients.

## Discussion

Conducting a comprehensive study using multi-omics data including bulk transcriptomics and genomics, scRNA-seq, snRNA-seq and spatial transcriptomics, we systematically unearthed the immunological implication and prognostic significance of CXCL12 in breast cancer. Of note, we promoted an efficacious prognostic signature based on CXCL12-related genes. Our findings provided a novel accurate classification and therapeutic strategy for breast cancer patients.

As a member of CXC chemokines, CXCL12 is expressed constitutively and ubiquitously. CXCL12 is traditionally identified as a homeostatic chemokine, since it involves in several physiological processes such as hematopoiesis, embryogenesis, angiogenesis, neurogenesis, cardiogenesis, leukocyte homing, and by inducing activation and migration of hematopoietic progenitor and stem cells, endothelial cells, as well as most leukocytes (7). An upregulated expression of CXCL12 in general or a specific increase of one of the CXCL12 splice variants could be found in diverse diseases (16). It reveals that CXCL12 shows diverse activities depending on the disease and could also be classified as an inflammatory chemokine. CXCL12 mainly interacts with the seven-transmembrane receptors CXCR4 and ACKR3, also known as chemokine receptor RDC-1 or CXCR7. Of late, it has been also demonstrated that CXCL12 can interact with ACKR1. The binding of CXCL12 to its receptors induces several intracellular signaling pathways through divergent pathways initiating signals linked with chemotaxis, cell survival and proliferation, as well as gene transcription and increase of intracellular calcium (17). CXCL12 is one of the most prominent chemokines fostering tumor cell survival and proliferation in models of ovarian cancer (18). Analogously, lower expression level of CXCL12 is associated with improved survival and prolonged OS in esophagogastric, lung cancer and pancreatic ductal adenocarcinoma (11, 19). Nevertheless, in breast cancer, high expression of CXCL12 is related to a prolonged OS and DFS (10), probably owing to decreased metastasis of breast malignant cells as found in the preclinical models (20). The effects of CXCL12 and CXCL12-mediated signaling pathways in breast cancer are multifaceted and highly context-dependent, varying among different molecular subtypes, stages of tumor progression, and the microenvironmental context. S100A9-CXCL12 signaling activation could enhance cancer progression and trigger the expansion and accumulation of myeloid-derived suppressor cells (MDSCs), producing a tumor-permissive microenvironment and endowing resistance to ICB in BRCA1-mutant breast cancers (21). In breast cancer mice models, olaparib could increase the antitumor efficacy of chimeric antigen receptor modified T (CAR-T) cell therapy at least partially through suppressing MDSC migration inhibiting the CXCL12-CXCR4 axis (22).

The combination of spatial transcriptomics and snRNA-seq or scRNA-seq serves as a pivotal component to link the pathological phenomes of human tissues with molecular alterations, which defines knowledge on spatiotemporal molecular medicine and *in situ* intercellular molecular communications. Spatial transcriptomics provides the molecular profiles and position information with high throughput, through spatial barcode microarrays for unbiased mapping of transcripts on the entire tissue section. We depicted the CXCL12 expression pattern in the histological sections to reveal the spatial location of CXCL12-expressing cells in breast cancer tissues. Using gene markers from the snRNA-seq, we identified CXCL12 was mainly expressed in the CAFs on the spatial image. However, our integrated scRNA-seq analysis further expanded the notion that CXCL12 was also expressed in a subset of endothelial cells and pericytes. Additionally, we speculated a significant role of CXCR4-CXCL12 axis in breast cancer, because of the extremely high expression of CXCR4 in immune cells, which represented the important immune-stromal interaction. Recent single-cell transcriptomics and functional co-culture experiments reported that CD26^+^ normal fibroblasts transition into pro-tumorigenic inflammatory CAFs which recruit myeloid cells through a CXCL12-dependent manner and induce tumor cell invasion via matrix-metalloproteinase activity in breast cancer (23).

A comprehensive interrogation of the prognostic implication of CXCL12 in breast cancer is eagerly needed. We found out that the expression level of CXCL12 was lower in breast cancer tissues compared with that in normal breast tissues. The decreased CXCL12 expression level was related to poor prognosis in the public breast cancer dataset and our validation cohort. These results revealed that absence of CXCL12 could be linked with the development of breast cancer. By performing WGCNA analysis, we identified the red module containing 402 genes was the most correlated module of CXCL12. Subsequent univariate Cox regression and LASSO regression analyses in breast cancer samples of TCGA screened out 11 genes including *VAT1L, TMEM92, SDC1, RORB, PCSK9, NRN1, NACAD, JPH3, GJA1, BMP8B* and *ADAMTS2* as the candidate genes for prognostic model construction. Among these candidate prognostic genes, some have been reported to exert an essential role in breast cancer, but some of them were not studied deeply. For example, TMEM92 was reported to act as an oncogene to support malignant cells growth, invasiveness and motility through regulating the EMT relative proteins in breast cancer (24). Moreover, SDC1 could enhance triple-negative breast cancer (TNBC) progression by activating the c-src/FAK signaling pathways (25). Additionally, Abdelwahed et al. reported that PCSK9 secretion and its interaction with LDL receptor were inhibited by pseurotin A, which was identified as a novel suppressor of hormone-dependent breast cancer progression and recurrence (26). After calculating the risk score, breast cancer patients in the training set were divided into high- and low-risk groups based on the median value of the risk score. We found that patients in the high-risk group harbored a considerable worse prognosis. Furthermore, we validated the accuracy of this prognostic model in three additional breast cancer datasets, which showed significant reliability.

Tumors are often considered to be heterogeneous. Our analysis thus unraveled the differences between high/low-risk groups at transcriptional and genetic levels and tried to solve the underpinning mechanism for their distinct outcomes. Pathway enrichment analysis revealed that the high-risk group was enriched in myogenesis, TNFα signaling via NF-κB, estrogen response and fatty acid metabolism, while the low-risk group was enriched in E2F targets, G2M checkpoint, MTORC1 signaling and glycolysis. Redeployment of modules typically expressed in other cellular and developmental contexts seems to result in the majority of the heterogeneity observed across cancer cells. We investigated the recently defined cancer cell states which represents the basic underlying units of tumor transcriptional variability and found different features in two risk groups. The high-risk group showed higher signature scores of cycling, hypoxia, mesenchymal and pEMT, but lower signature scores of AC, basal and stress. It has bene reported that TMB acts as a numeric value or binarized feature can only partially predict therapeutic response, since previous reports identifying TMB as a predictor of ICB response for patients with melanoma (27). Interestingly, we found that TMB was elevated in the high-risk group and positively connected with the risk score. Moreover, combination of TMB and the CXCL12-related risk score exhibited an excellent performance on predicting patient survival.

Tumor occurrence and development are linked with not only the genetic and epigenetic variation of tumor cells, but also the TME, which mainly composed of tumor cells, stromal cells and infiltrated immune cells. Tumor cells could survive and escape from immune surveillance and drug interference under specific TME. The destiny of macrophages relies on different environmental conditions, which fuel polarization to any of the classically triggered pro-inflammatory M1 response or triggered M2 immune response. M1-polarized macrophages could be induced by microbial products or pro-inflammatory cytokines, and M2-polarized macrophages could be triggered *via* interleukin (IL)-4 and IL-13. The classically activated M1-like macrophages exert an important role in anti-bacterial and anti-tumor immune responses, while the alternatively activated M2-like macrophages mainly participate in would healing, angiogenesis and immune suppression (28, 29). Previous studies have reported that the enrichment of M2-like macrophages indicates an inferior prognosis in breast cancer (30, 31). By CIBERSORT algorithm, we found high-risk group patients had significantly higher infiltration level of immunosuppressive M2-like macrophages, and lower infiltration level of CD8^+^ T cells and activated NK cells which were the main immune subsets exerting antitumor roles. The expression levels of immune checkpoint genes and antitumor infiltrating immune cells decreased in the high-risk group, implying holistic damage to immune functions. Moreover, our integrated analysis at single-cell resolution further demonstrated the risk score was positively correlated with M2-like macrophages. Thus, the elimination of M2 macrophages or their progenitors could represent a potential therapeutic strategy for high-risk group patients.

Owing to the inter- and intra-tumor heterogeneity, patients respond differently to treatments, contributing to treatment failure and occurrence. Therefore, we screened out several potential anticancer drugs for different group of breast cancers. IC50 of docetaxel was positively correlated with the risk score, indicating docetaxel was more sensitive to low-risk group patients. Immunotherapy has revolutionized therapeutic strategies for cancers, but most patients with solid neoplasms fail to respond to immunotherapy. In addition, the response to immunotherapy was also investigated in two groups. We found the high-risk group which showed higher level of TMB, had a prolonged survival in the immunotherapy cohort. Nevertheless, the exact function of above treatments needs to be further confirmed by future prospective studies.

Although our CXCL12-related prognostic model exhibited excellent performance in both training and validation cohorts, there are still several limitations. First, the mechanisms of action of CXCL12 in breast cancer are necessary to be validated *in vivo* and *in vitro*. Second, it might inevitably lead to some biases to some extent, because the patients were retrospectively recruited. Thus, additional validations in more multicenter randomized controlled trials with high quality, large sample size, as well as adequate follow-up are further required.

## Conclusions

Here, we comprehensively interrogate the role of CXCL12 in breast cancer by integrative multi-omics analyses. We then constructed and validated a CXCL12-related prognostic signature. There were significant differences between high- and low-risk groups in terms of prognosis, clinical characteristics, enrichment pathways, TMB, immune infiltration and treatment responses, which could help us to better understand the immunological implication and prognostic significance of CXCL12 in breast cancer, and establish novel directions for further exploration and research.

## Data availability statement

The original contributions presented in the study are included in the article/**Supplementary Material**. Further inquiries can be directed to the corresponding authors.

## Ethics statement

Written informed consent was obtained from the individual(s) for the publication of any potentially identifiable images or data included in this article.

## Author contributions

BL, S-RS, and J-PY contributed to the conception and design of this study. Z-JG and ZF collected and analyzed the data. Z-JG drafted the original manuscript. Z-JG and ZF polished and revised the manuscript. This manuscript has been read and approved by all authors.

## Supplementary material

Supplementary Figure 1 Correlation between CXCL12 and genetic as well as immunological features in breast cancer. **(A, B)** Scatter plot showing the correlation between CXCL12 and aneuploidy and MSI score in TCGA cohort. **(C)** Waterfall plot represents the mutation distribution of the most frequently mutated genes in CXCL12^high^ and CXCL12^low^ patients. **(D)** Bubble plot showing the relationship between CXCL12 and the proportion of 22 immune cells estimated by CIBERSORT in the TCGA breast cancer cohort.

Supplementary Figure 2 Prognostic potential of CXCL12 in breast cancer. **(A–E)** Kaplan-Meier survival analyses were performed on the relationship between CXCL12 and OS in the luminal A/B, normal-like, basal-like and HER2-enriched subtypes of breast cancer in the METABRIC cohort.

Supplementary Figure 3 Screening of CXCL12-related genes and validation of the CXCL12-related prognostic signature in breast cancer. **(A)** Coefficient profiles in the LASSO regression model. **(B)** Cross-validation for tuning parameter selection in the LASSO regression. **(D)** Kaplan-Meier survival analysis was performed on the relationship between the risk score and OS using the GSE21653 validation cohort. **(E)** The rank of risk scores in the GSE21653 validation cohort. **(F)** Survival status in the GSE21653 validation cohort. **(G)** Time-dependent ROC curve analysis of the prognostic model (1-, 2-, 3-, and 5-year) in the GSE21653 validation cohort. **(H)** Kaplan-Meier survival analysis was performed on the relationship between the risk score and OS using the GSE61304 validation cohort. **(I)** The rank of risk scores in the GSE61304 validation cohort. **(J)** Survival status in the GSE61304 validation cohort. (K) Time-dependent ROC curve analysis of the prognostic model (1-, 2-, 3-, and 5-year) in the GSE61304 validation cohort.

Supplementary Figure 4 Correlation between clinical characteristics and the risk score in breast cancer. **(A)** Risk scores of different tumor stages of breast cancer in the TCGA breast cancer cohort. **(B–D)** Kaplan-Meier survival analyses were performed on the relationship between the risk score and OS in the STAGE I, II and III of breast cancer in the TCGA breast cancer cohort. **(E)** Risk scores of different molecular subtypes of breast cancer in the TCGA breast cancer cohort. **(F–I)** Kaplan-Meier survival analyses were performed on the relationship between the risk score and OS in the luminal A/B, HER2-enriched and basal-like subtypes of breast cancer in the TCGA breast cancer cohort.

Supplementary Figure 5 Correlation between the risk score and pathway activities. **(A–C)**. GSEA analysis showing the up-regulated pathways in the high-risk group. **(D, E)**. GSEA analysis showing the up-regulated pathways in the low-risk group.

Supplementary Figure 6 scRNA-seq analysis of the tumor immune microenvironment features of breast cancer based on the CXCL12-related prognostic signature. **(A, B)**. UMAP plot showing the major cell subpopulations of high- and low-risk breast tumors. **(C–E)** UMAP plot showing the expression of CXCR4, ACKR3 and ACKR1 in breast tumors.

Supplementary Figure 7 High- and low-risk group patients differ in drug sensitivity. **(A–E)**. Boxplots showing the comparison of IC50 of drugs between high- and low-risk groups, and scatter plots showing the correlation between the IC50 of drugs and the risk score in the TCGA breast cancer cohort.
