# Supplementary figures and images for: Integrative multi-omics analyses unravel the immunological implication and prognostic significance of CXCL12 in breast cancer
Source: Front Immunol. 2023 Jul 26;14:1188351. doi: 10.3389/fimmu.2023.1188351 (PMC10410148; doi:10.3389/fimmu.2023.1188351)

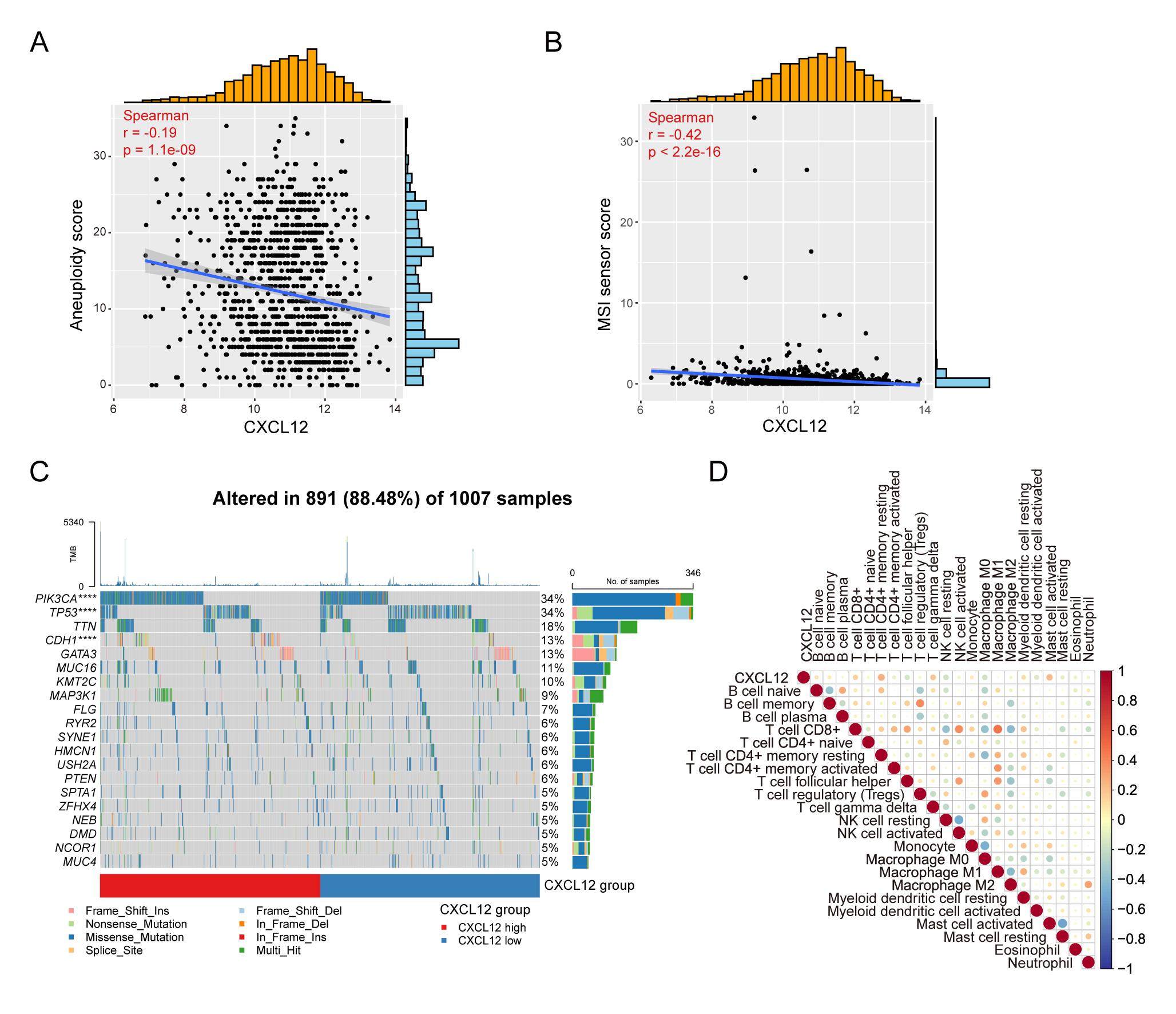

Supplement: Supplementary Figure 1 — Correlation between CXCL12 and genetic as well as immunological features in breast cancer. (A, B) Scatter plot showing the correlation between CXCL12 and aneuploidy and MSI score in TCGA cohort. (C) Waterfall plot represents the mutation distribution of the most frequently mutated genes in CXCL12high and CXCL12low patients. (D) Bubble plot showing the relationship between CXCL12 and the proportion of 22 immune cells estimated by CIBERSORT in the TCGA breast cancer cohort. [file Image_1.tif]

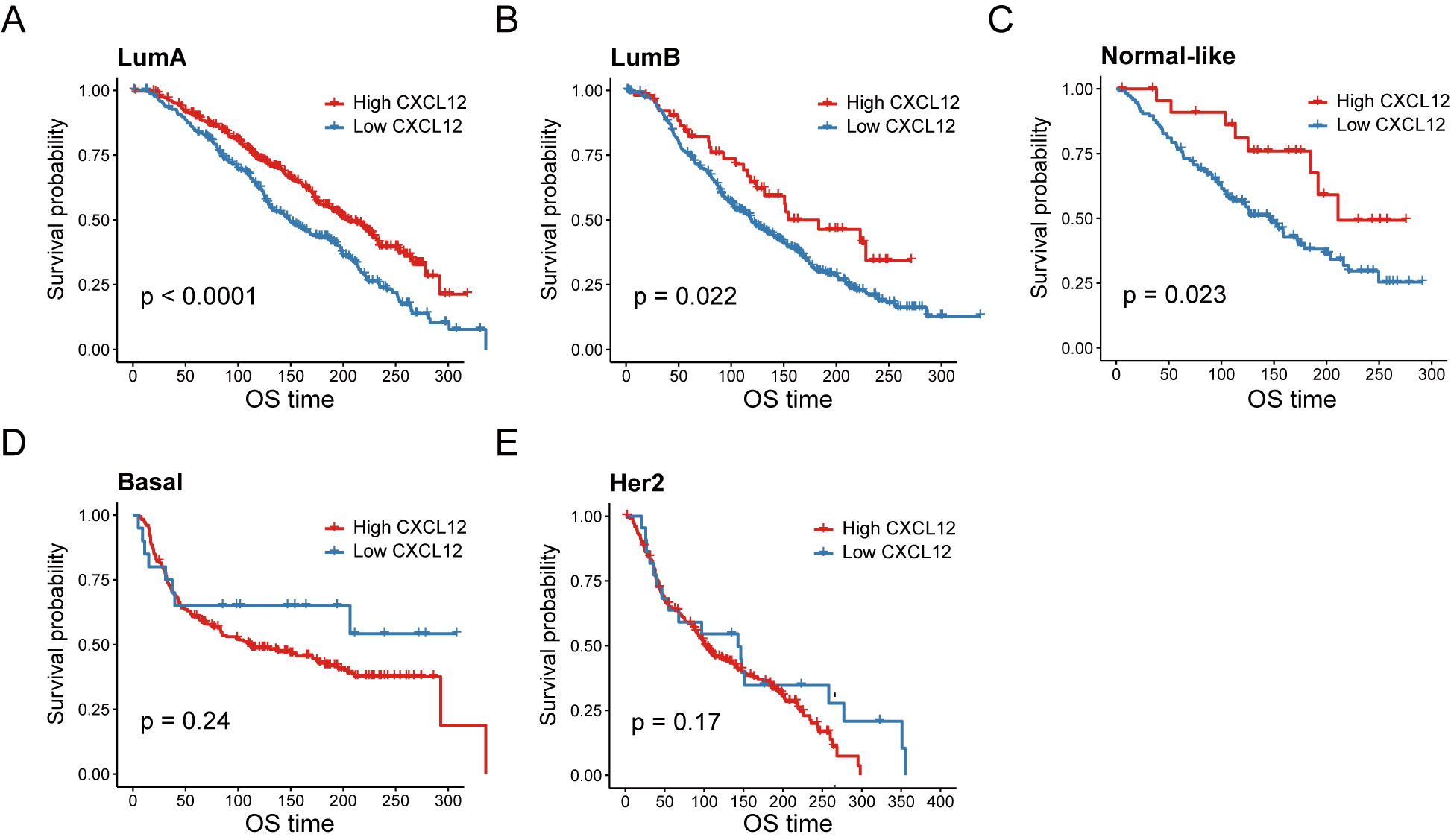

Supplement: Supplementary Figure 2 — Prognostic potential of CXCL12 in breast cancer. (A–E) Kaplan-Meier survival analyses were performed on the relationship between CXCL12 and OS in the luminal A/B, normal-like, basal-like and HER2-enriched subtypes of breast cancer in the METABRIC cohort. [file Image_2.tif]

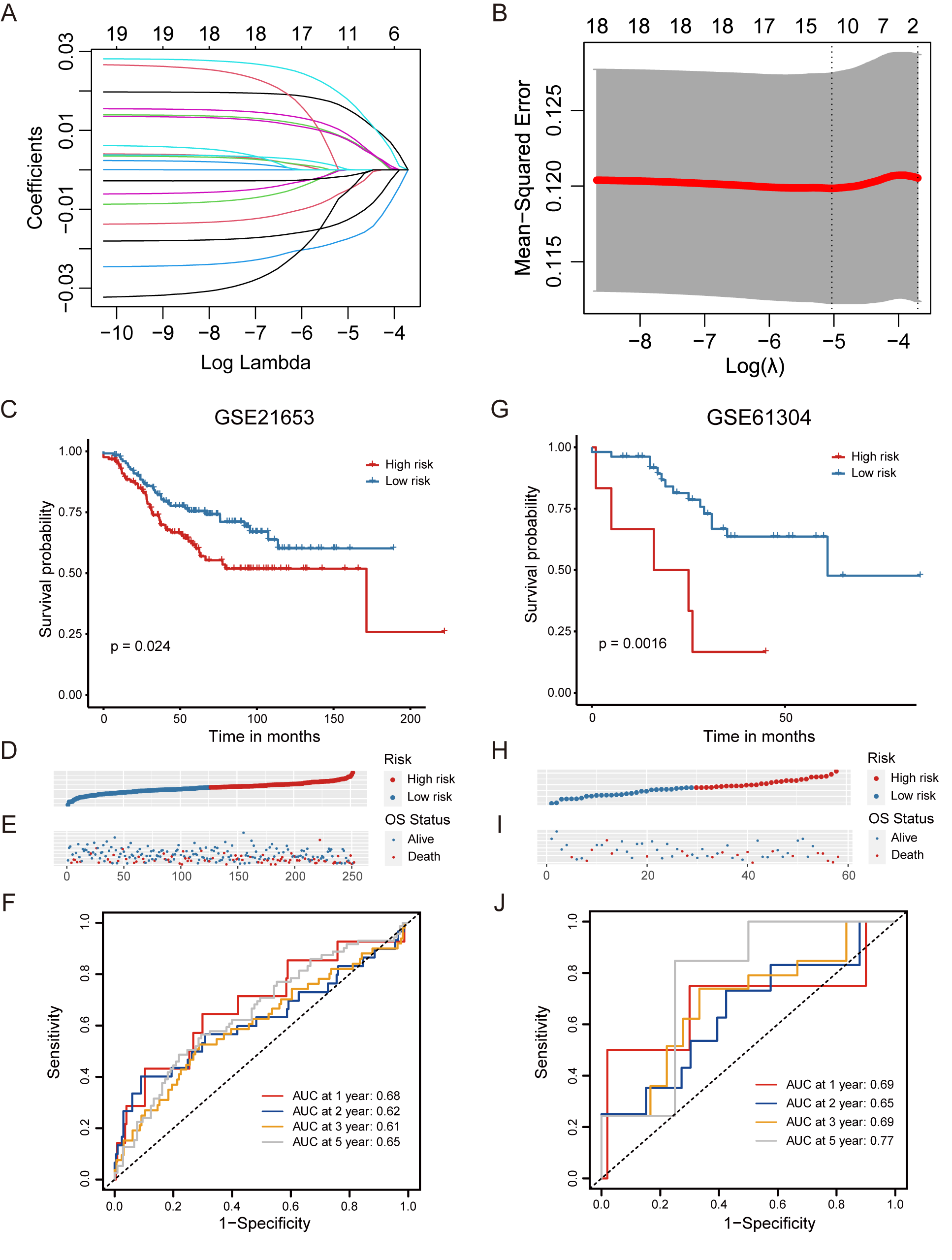

Supplement: Supplementary Figure 3 — Screening of CXCL12-related genes and validation of the CXCL12-related prognostic signature in breast cancer. (A) Coefficient profiles in the LASSO regression model. (B) Cross-validation for tuning parameter selection in the LASSO regression. (D) Kaplan-Meier survival analysis was performed on the relationship between the risk score and OS using the GSE21653 validation cohort. (E) The rank of risk scores in the GSE21653 validation cohort. (F) Survival status in the GSE21653 validation cohort. (G) Time-dependent ROC curve analysis of the prognostic model (1-, 2-, 3-, and 5-year) in the GSE21653 validation cohort. (H) Kaplan-Meier survival analysis was performed on the relationship between the risk score and OS using the GSE61304 validation cohort. (I) The rank of risk scores in the GSE61304 validation cohort. (J) Survival status in the GSE61304 validation cohort. (K) Time-dependent ROC curve analysis of the prognostic model (1-, 2-, 3-, and 5-year) in the GSE61304 validation cohort. [file Image_3.tif]

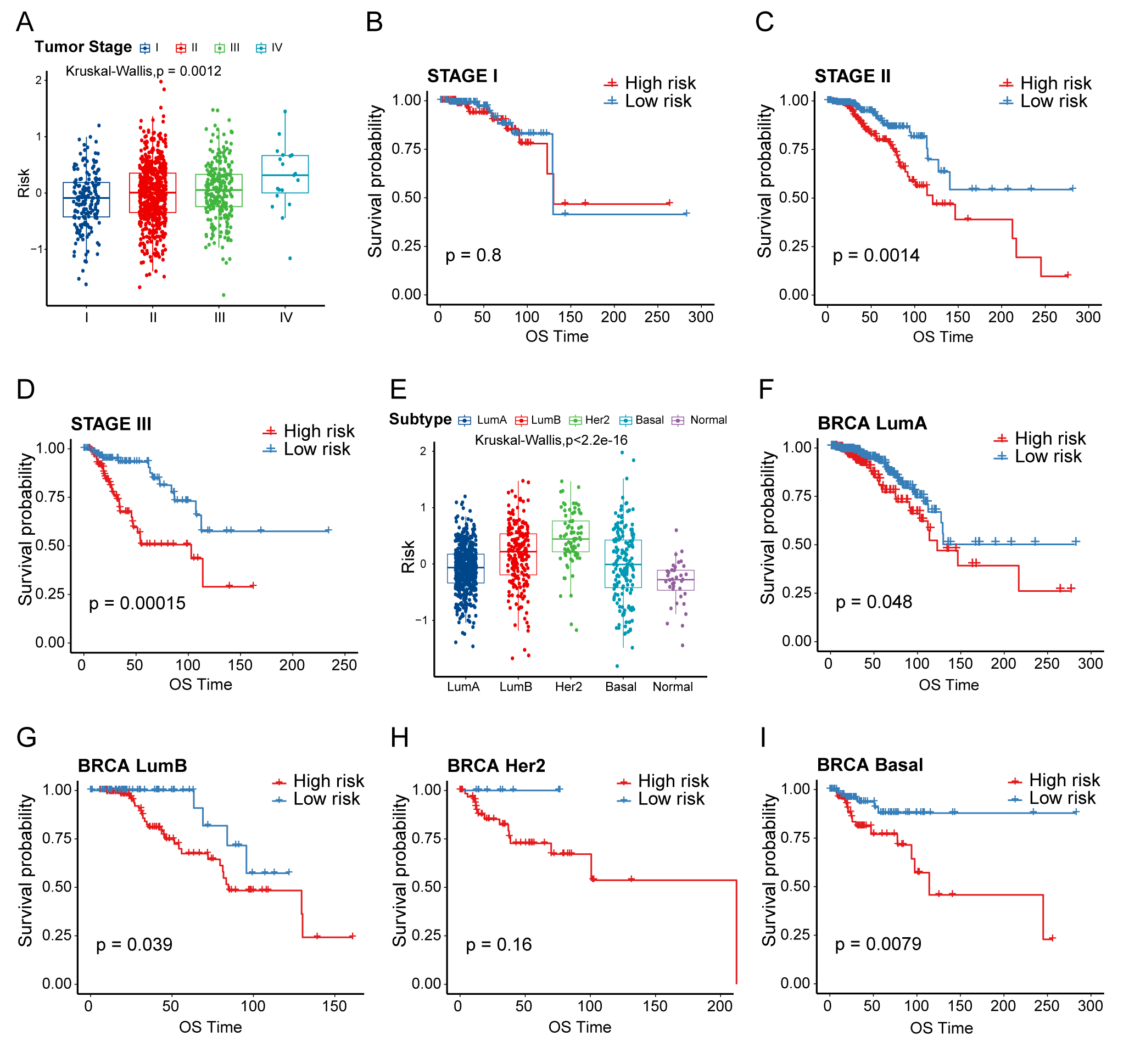

Supplement: Supplementary Figure 4 — Correlation between clinical characteristics and the risk score in breast cancer. (A) Risk scores of different tumor stages of breast cancer in the TCGA breast cancer cohort. (B–D) Kaplan-Meier survival analyses were performed on the relationship between the risk score and OS in the STAGE I, II and III of breast cancer in the TCGA breast cancer cohort. (E) Risk scores of different molecular subtypes of breast cancer in the TCGA breast cancer cohort. (F–I) Kaplan-Meier survival analyses were performed on the relationship between the risk score and OS in the luminal A/B, HER2-enriched and basal-like subtypes of breast cancer in the TCGA breast cancer cohort. [file Image_4.tif]

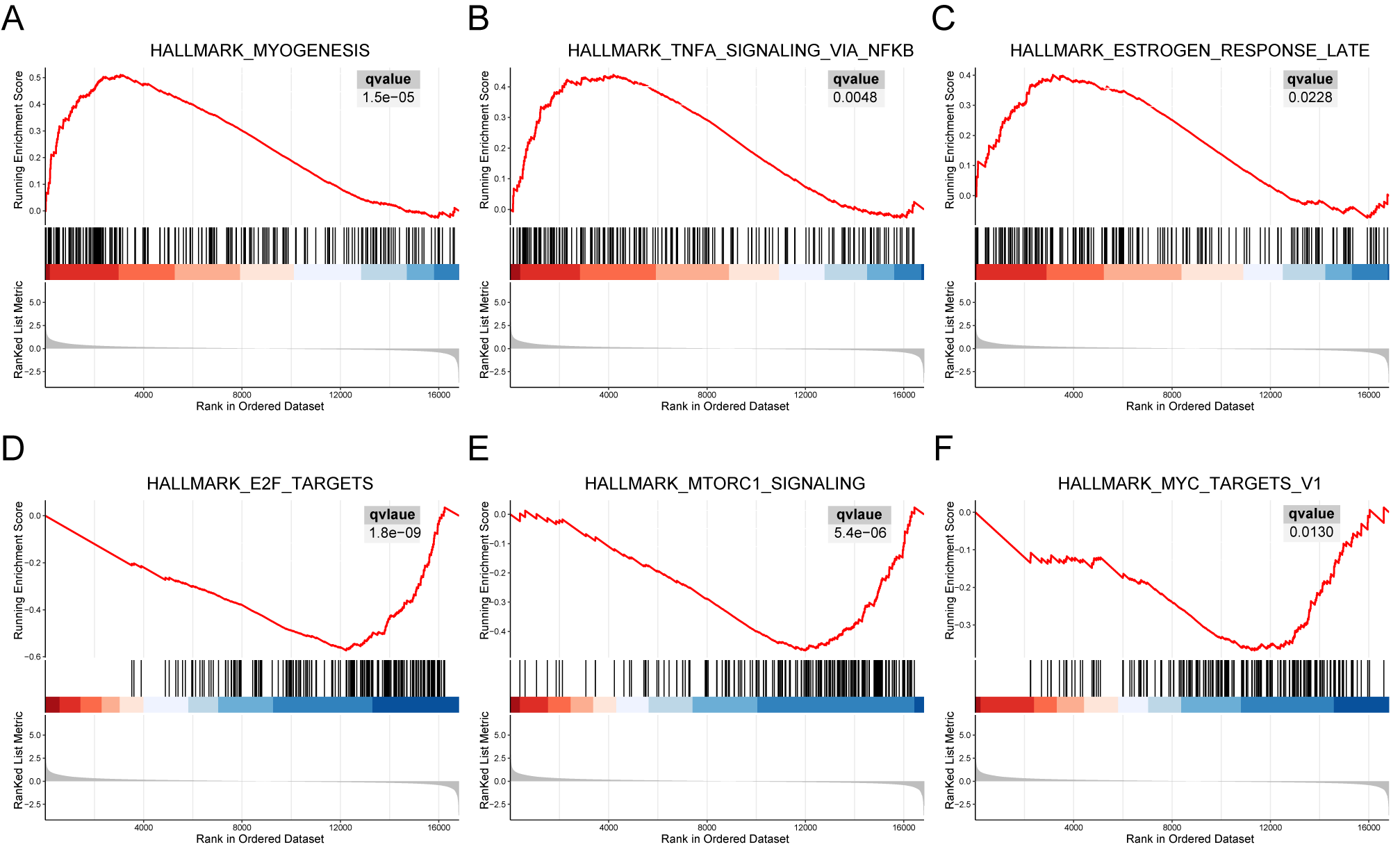

Supplement: Supplementary Figure 5 — Correlation between the risk score and pathway activities. (A–C). GSEA analysis showing the up-regulated pathways in the high-risk group. (D, E). GSEA analysis showing the up-regulated pathways in the low-risk group. [file Image_5.tif]

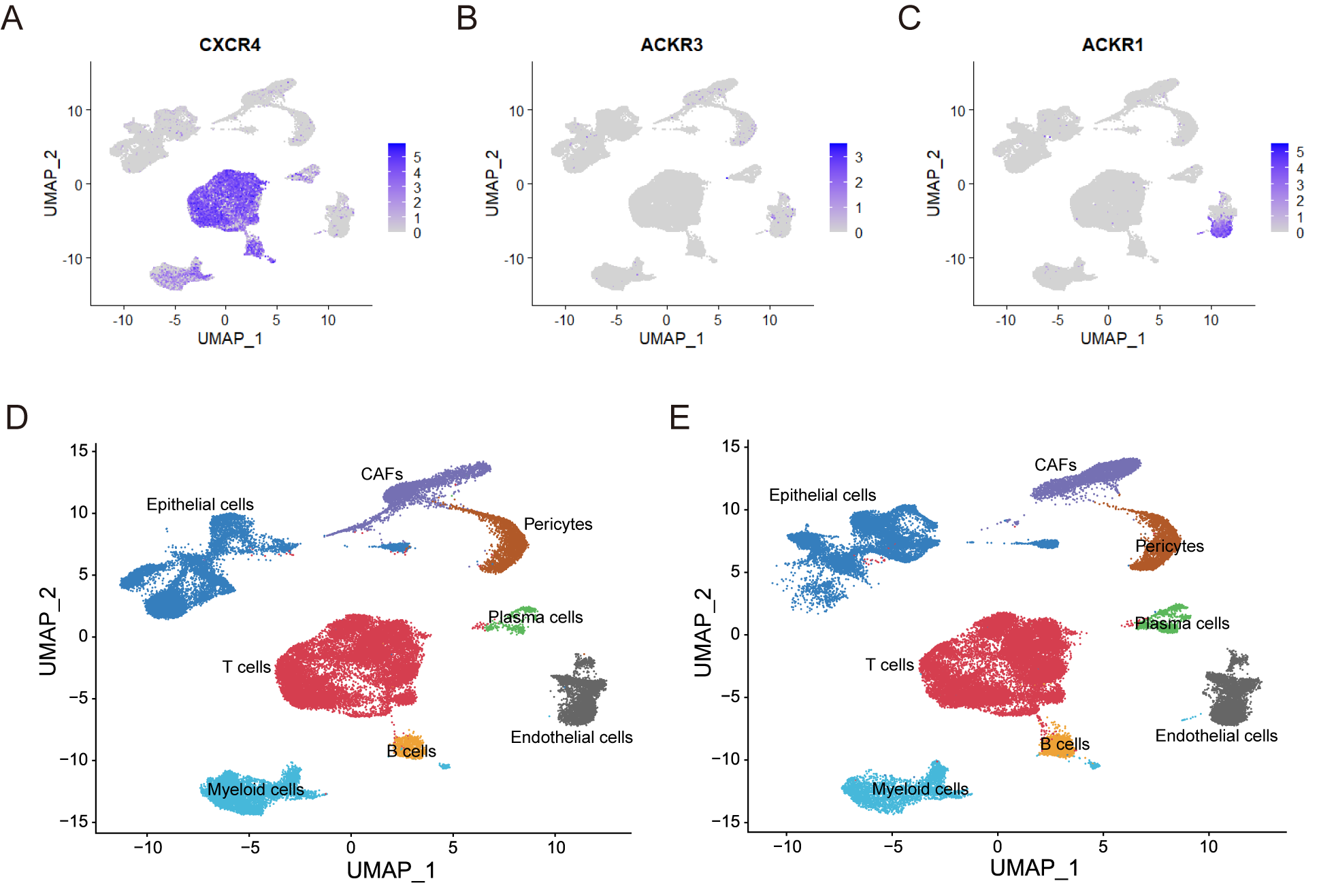

Supplement: Supplementary Figure 6 — scRNA-seq analysis of the tumor immune microenvironment features of breast cancer based on the CXCL12-related prognostic signature. (A, B). UMAP plot showing the major cell subpopulations of high- and low-risk breast tumors. (C–E) UMAP plot showing the expression of CXCR4, ACKR3 and ACKR1 in breast tumors. [file Image_6.tif]

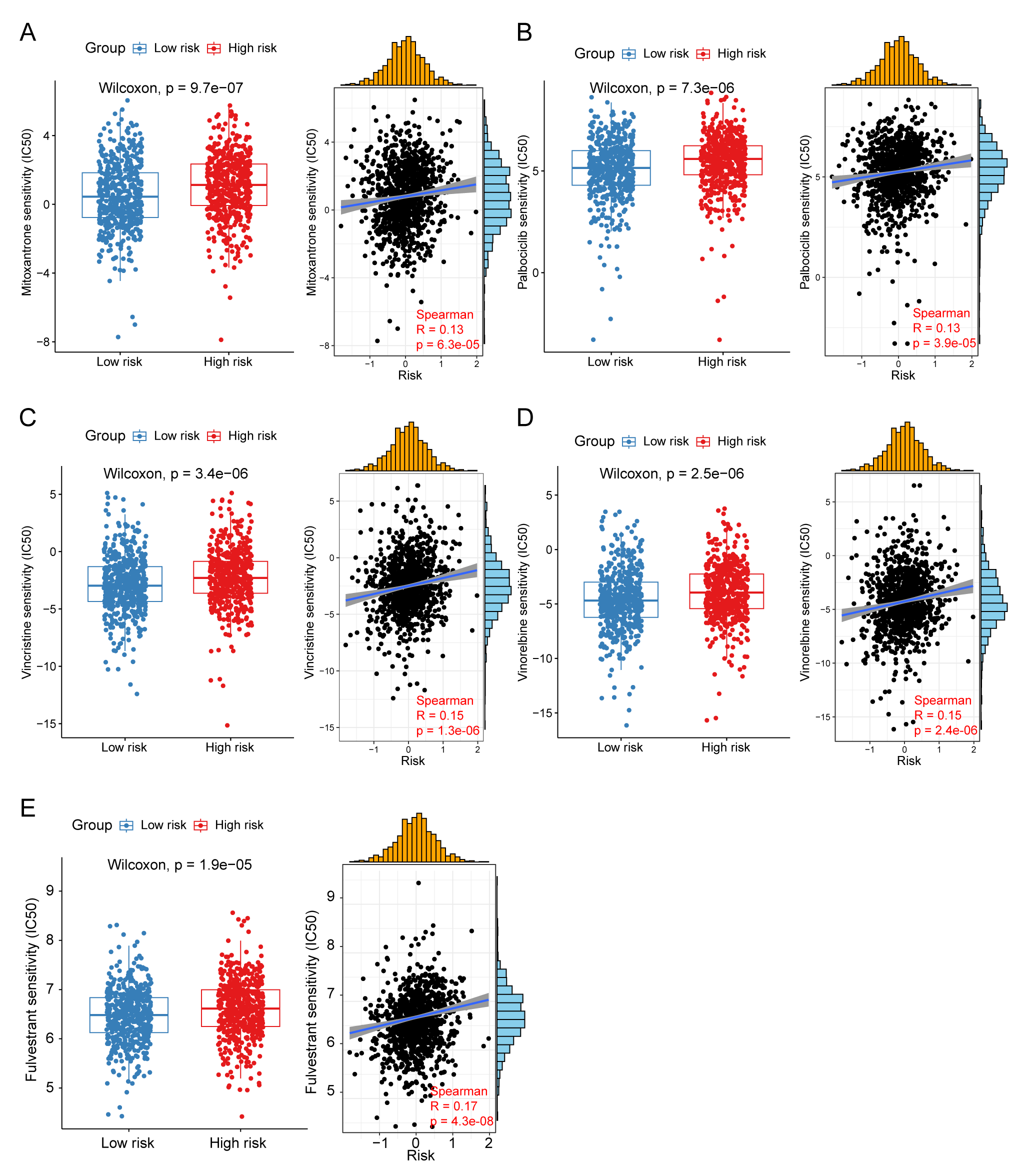

Supplement: Supplementary Figure 7 — High- and low-risk group patients differ in drug sensitivity. (A–E). Boxplots showing the comparison of IC50 of drugs between high- and low-risk groups, and scatter plots showing the correlation between the IC50 of drugs and the risk score in the TCGA breast cancer cohort. [file Image_7.tif]
